# Tensile Pull-Out Behaviour and Global Sensitivity Analysis of Fastening Screws in Photovoltaic Aluminium Support Structures

**DOI:** 10.3390/ma19143070

**Published:** 2026-07-16

**Authors:** Jiahang Zhang, Qunyi Huang, Mo Chen, Zhiyu Wang, Abudureyimujiang Aosimanjiang

**Affiliations:** 1 College of Civil Engineering, Southwest Jiaotong University, Chengdu 610031, China; 2 Jiangsu Key Laboratory of Mechanical Analysis for Infrastructure and Advanced Equipment, Southeast University, Nanjing 211189, China; 3 College of Civil Engineering, Kashi University, Kashi 844006, China; 4 CREGC Architectural & Construction Engineering Co., Ltd., Chengdu 610083, China; 5 School of Architecture and Environment, Sichuan University, Chengdu 610065, China

**Keywords:** aluminium alloy PV support structures, fastening screw, pull-out capacity, finite element modelling, global sensitivity analysis

## Abstract

Fastening screw connections in aluminium alloy photovoltaic (PV) support structures are highly susceptible to premature failure under wind-induced uplift suctions. This paper presents a systematic experimental, numerical, and probabilistic investigation into the tensile pull-out performance and interactive parameters of these joints. Static pull-out tests were performed to classify two typical failure mechanisms: thread stripping combined with base metal tearing (Failure Mode I) and screw shank tensile fracture (Failure Mode II). High-fidelity 3D finite element models incorporating explicit thread geometries were established, matching experimental curves with an error within 5% and accurately replicating micro-stress peeling contours. Furthermore, a multi-stage simulation loop integrating Latin Hypercube Sampling (LHS) and Response Surface Methodology (RSM) was implemented within a large-scale Monte Carlo framework to efficiently quantify parameter sensitivities under the elastic limit state. The global stochastic assessment reveals that the aluminium elastic modulus and RHS wall thickness are the primary positive controlling parameters, exhibiting high sensitivity coefficients of 0.44 and 0.40, respectively. Conversely, the profile width renders a significant negative sensitivity due to local out-of-plane flexing. These quantified indicators provide crucial structural optimisation directives for ensuring the safety of solar tracking and racking systems.

## 1. Introduction

**Figure 1:**
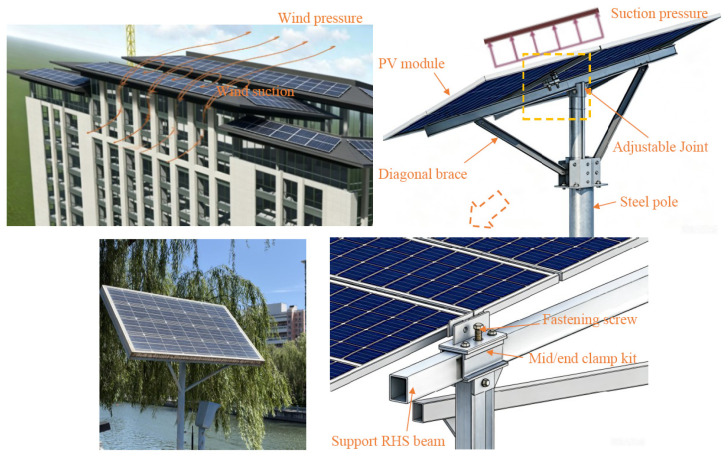
Typical single-pole adjustable bracket and screw-in clamp supporting PV module.

Aluminium alloy photovoltaic (PV) support structures have been comprehensively utilised in the solar industry owing to their lightweight nature, excellent strength-to-weight ratio, and superior corrosion resistance. Figure 1 illustrates a typical single-pole adjustable bracket and screw-in clamp supporting PV module system. This specific single-pole mounting bracket rack kit features an innovative orientation design, making it easy to adjust the angle among 0-15-30-45-60 degrees according to personal requirements. Such angle flexibility helps the solar panel get maximum sunshine exposure and generate more power efficiently across different seasons and geographic latitudes. From a structural perspective, the primary load-bearing skeleton of this racking system is composed of a closed section Rectangular Hollow Section (RHS) beam supported by a robust steel pole and reinforced via a diagonal brace. The photovoltaic (PV) modules are securely fastened to the top flange of the support RHS beam. This connection is firmly realised through a specialised mid/end clamp kit, which is anchored into the beam using high-strength fastening screws.

When these aluminium alloy PV support structures are widely installed on high-rise building roofs, they are exposed to extreme microclimate environments. A representative engineering case is illustrated in the architectural layout of Figure 1, which features a large-scale public building with a frame-double core tube structural configuration. This landmark structure rises to a total architectural height of 107 m, comprising multiple standard stories with a uniform height of 4.2 m per floor. Due to its significant elevation and standalone presence, the roof of this 107 m high-rise public building is directly subjected to amplified atmospheric boundary layer winds. Given the massive cumulative surface area of the roof-mounted PV modules, the entire racking system encounters severe aerodynamic wind loads. In real-world engineering applications, high-velocity winds sweeping over such tall roof boundaries generate a dominant negative wind pressure, commonly characterised as localised wind suction. Under the action of this intensive uplift suction pressure, a critical structural load transfer path is instantly activated; the uplifting aerodynamic force applied across the PV module plane is directly transmitted downward to the mid/end clamp kit. Subsequently, the mid/end clamp kit transfers the concentrated pulling load onto the fastening screw, subjecting the screw to a severe axial tensile or pull-out force.

Depending on the specific structural demands of these roof installations, both self-tapping and machine screws are currently widely adopted in the solar industry. Self-tapping screws enable rapid, direct anchoring on metal decks under low-load coplanar arrangements. Conversely, machine screws feature uniform, finer threads tailored for precision pre-tapped holes, delivering higher load-bearing capacity against heavy wind suction. The standardised installation of machine screws requires precise hole alignment, clockwise tightening, and calibrated torque control to prevent over-tightening. Given that adjustable, single-pole triangular configurations on high-rise roofs face extreme wind-induced tensile forces, maximising connection stability is paramount. If the induced wind uplift force exceeds the ultimate capacity of the joint during actual operation, the fastening screw will be abruptly pulled out from the RHS beam. Consequently, the PV modules will be instantly blown away, causing catastrophic destruction to the entire PV system and posing a severe threat to public safety in dense urban areas. Therefore, the connection integrity of the fastening screw directly dictates the safety and reliability of the solar PV system, making a rigorous experimental research and parametric analysis on its tensile pull-out bearing capacity of paramount engineering importance.

Currently, the majority of existing research on thin-walled components and RHS beam connections focuses on cold-formed thin-walled structures, providing a fundamental mechanical framework for evaluating localised tearing, buckling, and screw pull-out failure modes. For instance, Dubina et al. [1] evaluated the theoretical buckling strength degradation of cold-formed thin-walled steel members under coupled instability modes, while Kulatunga et al. [2] validated the compressive load-bearing capacity of columns with lipped channel sections using finite element (FE) models. For flexural elements, Ungureanu et al. [3] experimentally investigated back-to-back cold-formed steel channels under bending, revealing that spot-welded built-up sections exhibit comparable resistance to bolted ones while suppressing global buckling through enhanced local restraint. Regarding imperfection sensitivity, Zhang et al. [4] conducted experimental and FE analyses on built-up hot-rolled channel steel beams, quantifying the impact of bolt spacing and preload on flexural performance and identifying an optimal range (*L*/6 to *L*/4) for maximising structural efficiency. In terms of connections, Zhou et al. [5] examined the axial compression behaviour of double C-section partially encased composite columns, demonstrating that bolted stub columns outperform welded ones by 8.4% and that increased steel wall thickness improves load-carrying capacity by 16% under otherwise identical conditions. Recently, Ma et al. [6] comprehensively investigated the application of cold-formed thin-walled high-strength steel in PV brackets through testing 55 specimens, proposing verified bearing capacity formulas for both brackets and bolted connections.

Predicting the capacity of these thin-walled connections is addressed by several design standards. Detailed design formulas for predicting screwed fastener shear strength under tilting and bearing failure modes are included in Eurocode3 [7] and the Australian/New Zealand Standard [8], where the ultimate failure strength is determined by the lowest calculated value among different modes. Additionally, the US Aluminium Design Manual (ADM 2015) [9] accounts for the nominal tensile capacity of screw holes by explicitly considering parameters such as connection location, thickness, yield strength, ultimate strength of the aluminium profile, and screw head/washer diameters. Regarding specific fastener types, historical research has predominantly focused on self-tapping screws. Song [10] identified the “group effect” in self-tapping screw connections and introduced a screw spacing correction factor. Zeng et al. [11] found that experimental results align closely with US standard calculation methods and provided practical recommendations for pull-out formulas. From a theoretical perspective, Wang et al. [12] utilised ANSYS (Version 2019 R1) numerical simulations to study the connection performance of self-tapping screws, offering a foundational reference.

Advanced 3D modelling techniques have been widely applied to refine these simulations, though historical challenges persist regarding thread representation. Huynh et al. [13] developed detailed 3D FE models incorporating fracture criteria to simulate failure modes of cold-formed steel connections. However, these models failed to explicitly model screw threads, relying instead on artificial friction factors that limit applicability across varying sheet thicknesses or screw types, and their fracture criteria neglected the critical influence of the Lode angle on thin plate fracturing. To overcome these limitations, Wang et al. [14] investigated the tension performance of cold-formed square tube joints with self-tapping screw connections, noting that explicit geometric representation of screw shank and head is essential for accurately predicting tensile capacity without resorting to arbitrary friction coefficients; Wang [15] examined the eccentric compression behaviour of L-shaped columns fabricated by thin-walled square steel tubes based on self-drilling screw connections, highlighting the influence of screw configuration on global stability and local deformation; and Zhou et al. [16] proposed a simplified nonlinear explicit dynamic FE model using shell and connector elements to capture the full-range behaviour of screwed connections—including pull-out, screw shear and clip-angle buckling—while eliminating the need for artificial friction factors or idealised thread idealisation. Additionally, the latest research [17] introduced a deep transfer learning framework combined with deep neural networks to rapidly predict multi-dimensional deformations of threaded connections under complex loading conditions, drastically reducing computational cost to 0.14% of traditional FEA processing time while achieving a high prediction accuracy of up to 96.6% across various bolt geometries.

Despite these advancements, existing research has predominantly focused on cold-formed steel and self-tapping screws under shear loading, leaving a noticeable research gap regarding the tensile pull-out performance of fastening screws in aluminium alloy configurations. In particular, there is a lack of comprehensive studies exploring the interactive failure mechanisms between machine clamping screws and thin-walled aluminium profiles under wind-induced uplift forces, as well as a lack of efficient computational frameworks to analyse their parameter sensitivities under construction overloads. To bridge these gaps, this paper presents a rigorous experimental and numerical investigation into the tensile performance of fastening screw connections in aluminium alloy RHS beams. First, a series of static pull-out tests were conducted on clamping screw connections to aluminium alloy RHS beams. Based on the experimental outcomes, two typical failure modes of the RHS profiles and clamping screws were identified, the force-displacement relationship characteristics under axial loading were established, and a preliminary analysis of the key influencing parameters was performed. Subsequently, high-fidelity three-dimensional finite element (FE) models explicitly incorporating the thread interactions were developed using ANSYS. These numerical models enabled a meticulous analysis of the local stress contours, yielding propagation, and contact behaviours during the pulling process. Furthermore, to execute the reliability and global sensitivity analysis under construction overloads without incurring prohibitive computational costs, a hybrid reliability simulation strategy was implemented within the ANSYS Probabilistic Design System (PDS). The framework seamlessly integrates the Latin Hypercube Sampling (LHS) technique with the Response Surface Methodology (RSM) through a multi-stage simulation loop. Based on this robust stochastic framework, the individual and coupled effects of various material properties and geometric configurations of both the fastening screw and the RHS beam on the ultimate bearing capacity were thoroughly evaluated, followed by an in-depth global sensitivity analysis. Distinguished from conventional deterministic engineering case studies, the fundamental scientific contribution of this study lies in establishing a multi-scale decoupled probabilistic mapping framework that bridges localised microscopic thread-stripping damage mechanisms with macro-structural stochastic reliability boundaries. While the individual experimental or numerical techniques are established, their highly nonlinear integration, specifically coupling LHS with RSM, is conducted to quantify the boundary sensitivity thresholds of thin-walled aluminium joints under highly localised stress fields. This presents a systematic analytical methodology that provides transferable scientific insights for other thin-walled metal fastenings.

## 2. Experimental Study and Results

### 2.1. Materials and Specimen Configurations

**Figure 2:**
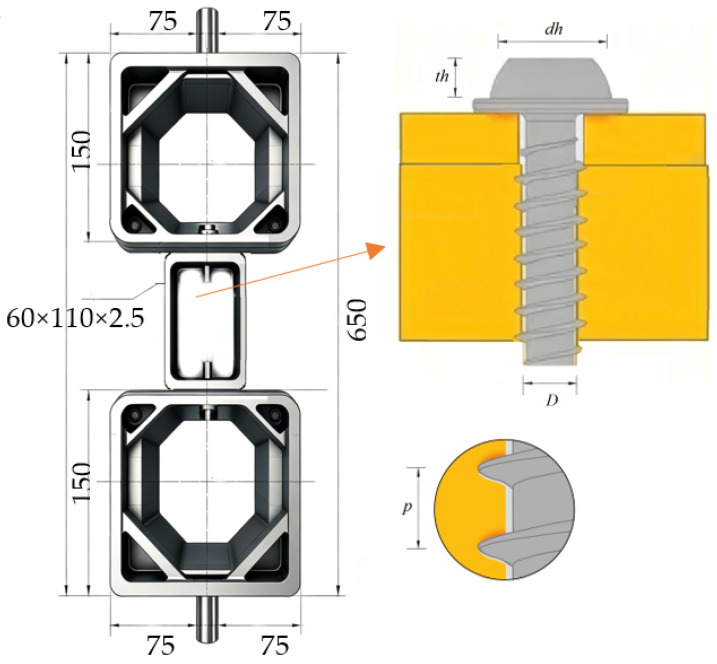
Illustration of RHS screwed to clamping element.

A series of joint specimens consisting of aluminium alloy Rectangular Hollow Section (RHS) profiles and fastening screws were prepared to investigate the tensile pull-out performance. The aluminium alloy RHS profiles utilised across all specimens were fabricated from 6063-T6 aluminium alloy, with a uniform cross-sectional dimension of 60 mm × 110 mm × 2.6 mm. Two distinct types of fasteners were evaluated in this study, namely self-tapping screws (SS) and machine screws (MS). As detailed in Table 1, the geometric variables of these fasteners encompassed a comprehensive range of dimensions: the nominal shank diameter (*D*) varied from 3.58 to 7.90 mm, the thread pitch (*p*) ranged from 0.8 to 1.6 mm, the screw head width (*d*_h_) spanned from 6.8 to 15.7 mm, and the screw head thickness (*t*_h_) ranged from 2.7 to 5.9 mm. The detailed schematic layout of the assembly is illustrated in Figure 2, and the comprehensive test matrix with specific specimen designations is compiled in Table 1.

The installation and tightening procedures for the SS and MS joints followed strict standardised configurations to minimise experimental discrepancy. For the SS specimens, the fasteners were driven directly into the flange of the 6063-T6 RHS profile using a calibrated electric screwdriver. The self-drilling tip of the SS penetrated the aluminium wall and dynamically formed its own mating internal threads until the screw head was fully seated. Conversely, the installation of the MS specimens required a two-step sequential process. First, a high-precision pre-drilled hole was executed on the flange of the RHS profile using a drill bit with a diameter matching the minor thread diameter of the MS. Second, internal threads were accurately generated within the pre-drilled hole utilising a hand tap, after which the MS was manually engaged into the tapped hole and tightened clockwise. For both joints, a digital torque wrench was employed to apply a precise, calibrated seating torque according to manufacturer specifications, ensuring stable clamping without causing premature micro-stripping of the thin-walled aluminium internal threads prior to axial testing. The selection of the geometric configurations in Table 1 was strictly based on mainstream commercial solar PV racking product standards and typical engineering practices for high-rise roof installations. For each joint variant, three identical specimen repetitions were fabricated and tested under identical laboratory conditions to ensure statistical reproducibility. The experimental parameters (the ultimate peak load *F*_u_, and its corresponding displacement *δ*_u_ and secant stiffness *K*_u_) compiled in Table 1 represent the calculated average values from these valid repetitive tests, with the maximum standard deviation for *F*_u_ remaining below 4.2%, demonstrating high experimental reliability.

### 2.2. Test Setup and Loading Procedure

**Figure 3:**
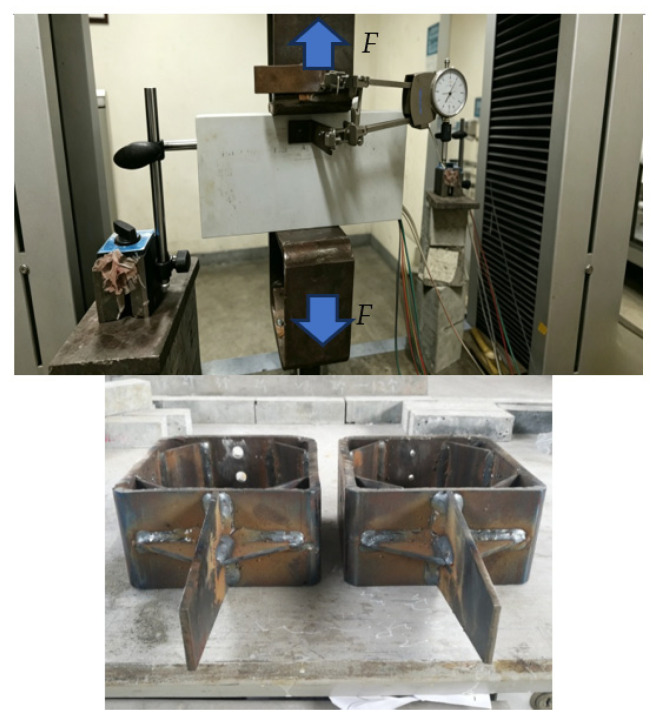
Schematic of test photos. (Blue arrow-illustration of applied force and reaction).

The axial pull-out tests were executed using a Shimadzu AGS-X 100kN servo-electronic universal testing machine (Shimadzu Corporation, Kyoto, Japan) equipped with an integrated data acquisition system. The testing system possesses a maximum rated capacity of 100 kN with a load measurement accuracy within ±0.5% across a range of 0.2% to 100% of the maximum capacity, utilised via a high-precision spoke-type load cell. As illustrated in the schematic layout (Figure 2) and the actual test photos (Figure 3), a specialised rigid dual-symmetric clamping assembly was developed to support the thin-walled aluminium components. The setup consists of two thick-walled steel frames (with a cross-section of 150 mm × 150 mm) configured at the top and bottom. These rigid frames were anchored to the loading heads of the universal testing machine through solid 75 mm diameter steel pins, sandwiching the 6063-T6 RHS specimen in between to apply a balanced tensile force (*F*) across the dual fastening screw joints. To prevent dynamic effects, a quasi-static displacement-controlled loading scheme was adopted at a constant rate of 0.5 mm/min until catastrophic connection failure occurred.

Directly utilising the gross crosshead displacement recorded by the testing machine to establish the load–displacement relationship is inherently unreliable. The gross displacement encompasses not only the actual slip of the screw relative to the screw hole but also includes the localised out-of-plane deformation of the RHS flange surface, the elastic deformation of the testing machine frame, and the compliance of the heavy steel fixtures. To accurately isolate the genuine pull-out displacement of the fastening screw within the aluminium hole, a refined hybrid measurement system incorporating both an extensometer with a 25 mm range and a dial gauge with a 5 mm range was implemented. The extensometer was positioned to bridge the screw head and the RHS flange surface, capturing the relative movement between the two connection surfaces. Simultaneously, the dial gauge was mounted to measure the localised out-of-plane deflection of the RHS profile wall. By subtracting the structural deflection recorded by the dial gauge from the total deformation obtained via the extensometer, the true, net tensile pull-out displacement (*δ*) of the screw inside the hole was successfully determined for subsequent characteristic analysis.

### 2.3. Test Results and Discussion

#### 2.3.1. Experimental Failure Modes

**Figure 4:**
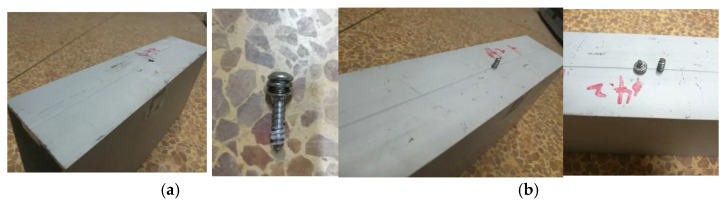
Experimental failure mode. (**a**) Failure mode I. (**b**) Failure mode II.

Based on the axial pull-out test observations illustrated in Figure 4, two primary and distinct failure modes were categorised depending on the relative stiffness and strength combinations between the fasteners and the aluminium alloy profiles:

- Failure Mode I (Thread Pull-out and Base Metal Tearing): As shown in Figure 4a, the fastening screw itself exhibited no observable structural damage or thread deformation. Instead, the internal threads formed within the RHS profile hole were severely stripped and sheared off, accompanied by a distinct localised out-of-plane residual protrusion around the perimeter of the screw hole on the flange surface. Upon reaching the ultimate tensile capacity, the connection failed abruptly. This mode occurs because the inherent shear capacity and cross-sectional stiffness of the screw are substantially greater than those of the thin-walled aluminium wall, shifting the primary damage to the base metal.
- Failure Mode II (Screw Shank Torsional-Tensile Fracture): Illustrated in Figure 4b, this failure mode was characterised by the catastrophic tensile fracture of the screw shank at the junction beneath the screw head. The fractured shank remained embedded within the RHS wall, while a mild residual out-of-plane deformation was still visible on the aluminium surface around the hole. Similar to Mode I, the load dropped instantly after reaching the peak capacity. In this case, the ultimate bearing capacity and geometric stiffness of the screw are closely matched with or slightly lower than those of the engaged base metal threads, causing concurrent damage to both components until the screw fractured.

#### 2.3.2. Characteristics of F-Δ Curves

**Figure 5:**
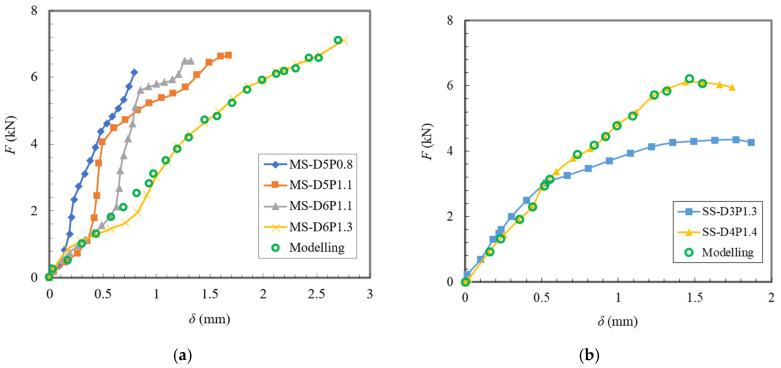
Plot of experimental and modelling *F*-*δ* curves. (**a**) Failure mode I. (**b**) Failure mode II.

The experimental *F*-*δ* responses for both failure modes are plotted in Figure 5. The curve characteristics exhibit a fundamental mechanical divergence between the two modes, particularly during the initial loading phase: (1) Initial adaptation and seating phase: a highly prominent non-linear seating or “compaction” stage was observed exclusively at the beginning of the *F*-*δ* curves for specimens exhibiting Failure Mode I (Figure 5a). This initial low-stiffness slip is explicitly caused by the localised micro-tearing and major plastic bending deformation of the thin-walled aluminium RHS profile around the screw hole under early tensile increments, allowing the threads to re-engage and re-seat. In sharp contrast, this feature is completely absent in Failure Mode II (Figure 5b), where the curve directly enters a linear-elastic stage. This is because the screw shank deformation dominates the compliance before fracture, with minimal severe macro-deformation occurring at the aluminium profile interface. (2) Elastic and softening regimes: following the initial phase, both modes transitioned into a stable elastic regime with constant stiffness, followed by a non-linear softening branch up to *F*_u_ due to cumulative plastic yielding. As shown by the modelling indicators in Figure 5, the high-fidelity FE numerical results accurately trace these stages, successfully validating the test curves for both SS and MS series.

#### 2.3.3. Parametric Influence on Experimental Results

As a fundamental baseline derived from Table 1, *D* primarily governs the geometric classification of the failure modes, where smaller values (e.g., *D* = 3.58 mm) are prone to Failure Mode II and larger values (e.g., *D* > 5.0) mm) shift the mechanism toward Failure Mode I. Beyond this geometric baseline, *p* and the intrinsic material strengths exert a more intricate influence on *F*_u_. For instance, when comparing specimens with identical *D* under Failure Mode I, a smaller *p* typically yields a higher *F*_u_ due to the increased number of engaged threads within the localised 2.6 mm thick aluminium wall. As exemplified by the MS-D5 series, reducing *p* from 1.1 mm to 0.8 mm resulted in a discernible increase in *F*_u_, as a finer thread optimises the continuous mechanical interlocking. Conversely, an overly large *p* reduces the interlocking thread count, thereby compromising the total shear resistance area of the internal aluminium thread cylinder. Furthermore, specimen groups failing under Failure Mode I displayed higher post-yield deformability but lower secant stiffness *K*_u_ (*F*_u_/*δ*_u_) compared to Failure Mode II. For example, Failure Mode I specimens exhibited substantial plastic bearing, leading to a prolonged softening branch with *δ*_u_ often exceeding 1.5 mm to 2.5 mm, which consequently reduced *K*_u_. In contrast, Failure Mode II specimens (such as the SS-D3P1.3 configuration) demonstrated a highly linear-elastic behaviour prior to sudden rupture, characterised by a significantly higher *K*_u_ but a much lower *δ*_u_, which typically remained below 1.5 mm. This reflects the linear-elastic dominance of the screw shank under pure axial tension prior to its sudden fracture.

Although these macroscopic trends are identifiable from the physical test matrix, the highly interactive mechanical behaviours, such as the localised stress distributions, the contact friction states at the micro-thread interfaces, and the individual sensitivity of each coupled geometric or material parameter, cannot be quantified solely through experimental surface measurements. This limitation highlights the necessity for a more rigorous numerical approach. Consequently, high-fidelity finite element models and advanced probabilistic simulations are implemented in the subsequent sections to thoroughly decouple these parameters and evaluate their global sensitivities on the tensile resistance.

To further elevate the analytical depth of the experimental repository, the obtained ultimate capacities were benchmarked against the nominal tensile capacities calculated using current mainstream structural design standards, specifically the US Aluminium Design Manual (ADM 2015) [9]. According to ADM 2015 [9], the nominal pull-out strength (*R*_n_) of the screw connection can be calculated as follows:

(1) $$R_{n}=\left\{\begin{array}{l} \Gamma \cdot t_{c}\cdot f_{a,u}\cdot \left(dh-D\right),\begin{array}{c} \;\mathrm{when}\;t_{c}/dh>0.5 \end{array}\\ \left(1.0+1.7t_{c}/dh\right)\cdot dh\cdot t_{c}\cdot f_{a,y},\;\mathrm{when}\;t_{c}/dh\leq 0.5 \end{array}\right.$$

where *t*_c_ represents the wall thickness of the aluminium alloy RHS and *f*_a,y_ and *f*_a,u_ are the yield strength and ultimate strength of the aluminium alloy RHS profile, respectively. The factor Γ depends on the location of the screw joint, being taken as 1.0 if the screw is located at the top profile flange and 0.7 if at the bottom.

The comparison reveals that ADM 2015 [9] generally overpredicts the tensile capacity of the joints across all tested configurations. For Failure Mode I (thread pull-out), the nominal code predictions overpredict the capacities by 12% to 52% (e.g., overpredicting MS-D5P0.8 by 14%). This severe overestimation arises because the standard code design relies on simplified uniform shear stress block assumptions over the total wall thickness, failing to account for the localised micro-clamping degradation, thread deformation, and friction loss under high out-of-plane flexing of the thin-walled 6063-T6 flange. Similarly, for Failure Mode II (shank fracture), ADM 2015 [9] overpredicts the capacities by 2% to 22% (e.g., overpredicting SS-D3P1.3 by 20%). This discrepancy indicates that, while standard engineering formulas consider pure tensile capacity governed by the screw net section area, they neglect the complex multi-axial stress states and localised stress concentrations at the screw thread root under realistic joint eccentricities, leading to non-conservative predictions for brittle shank fracture.

## 3. Numerical Modelling

### 3.1. Model Description

**Figure 6:**
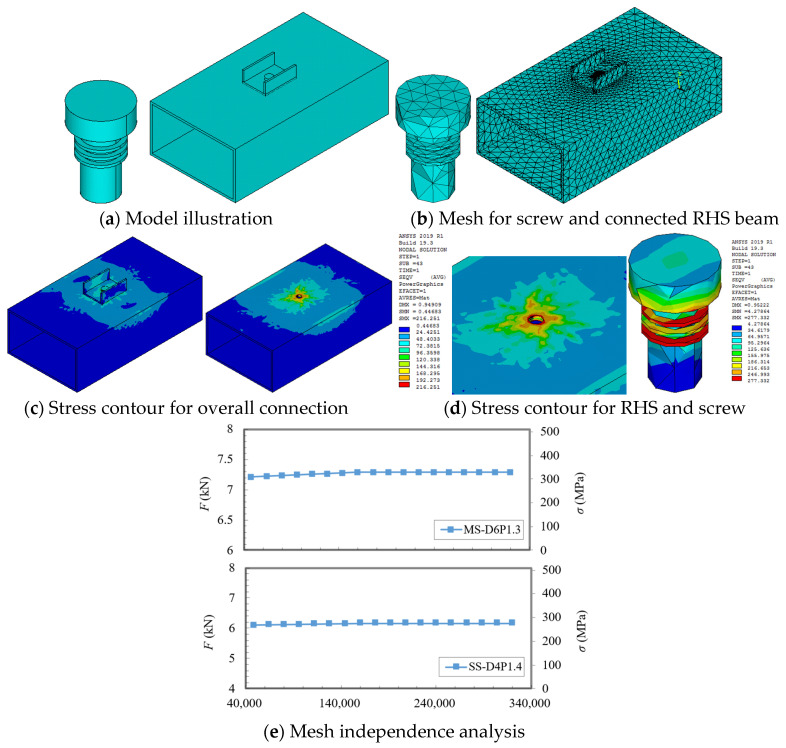
Finite element model, mesh plot, typical stress contours and mesh independence.

To accurately capture the localised stress distributions and highly non-linear geometric interactions at the micro-thread interface, a three-dimensional (3D) finite element (FE) model was established using ANSYS. As illustrated in the model schematic in Figure 6a, both the fastening screw and the connected aluminium alloy RHS beam incorporating the upper clamping channel features were modelled based on their exact experimental geometries. All structural components in the simulation were exclusively discretised using the SOLID185 element type, which is an eight-node 3D solid element defined by three translational degrees of freedom at each node and well-suited for simulating large deformations, plasticity, and complex contact behaviours. The mesh morphological layout of the assembly is presented in Figure 6b. To strike an optimal balance between computational precision and efficiency, a multi-zone mesh refinement strategy was strictly implemented. The complex geometric profiles of the helical threads on the fastening screw shank, as well as the mating internal thread contours within the RHS profile hole, were explicitly modelled and discretised with a highly dense, fine mesh. This localised refinement guarantees an accurate representation of the intense stress gradients and progressive yielding during the axial pull-out process. Conversely, for the regions of the RHS beam body situated further away from the centralised connection zone, a relatively coarser mesh density was adopted to substantially alleviate the computational overhead without compromising the global structural response.

### 3.2. Material Model

**Figure 7:**
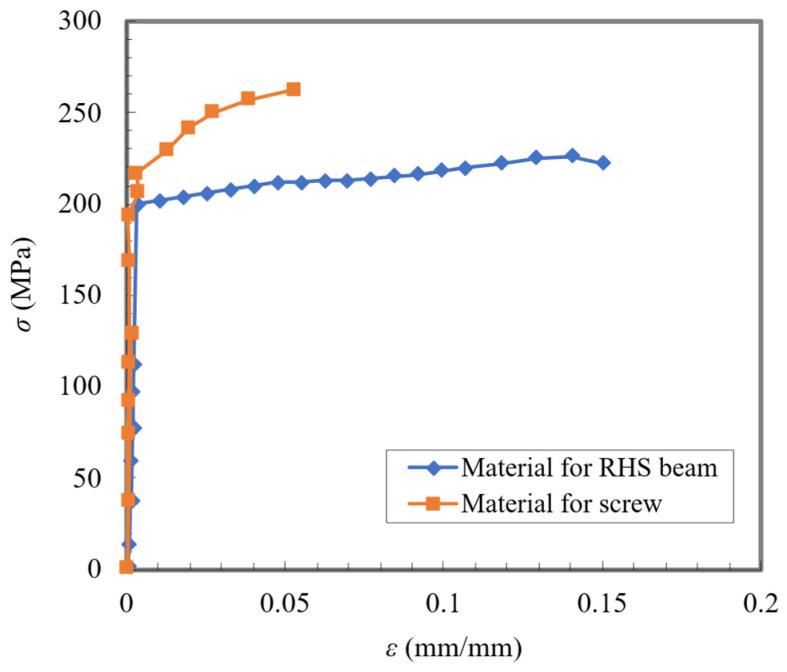
True *σ*-*ε* relation defined in numerical model.

The highly non-linear material responses and boundary interfaces were carefully defined to replicate the experimental physical behaviours. The true stress–strain (*σ*-*ε*) relationship for both the fastening screw and the aluminium alloy RHS beam in Figure 7 exhibit characterised continuous post-yield hardening regimes without a distinct yield plateau. Consequently, a Multilinear Isotropic Hardening (MISO) constitutive model was implemented for both materials. This framework tracks plastic multi-stage yielding by directly implementing a series of continuous data points extracted from the material calibration curves. For the elastic baselines, the fastening screw was assigned a steel elastic modulus (*E*_s_) of 206,000 MPa and a yield strength (*f*_s,y_) of 220 MPa. The 6063-T6 aluminium alloy RHS profile was defined with an (*E*_a_) of 70,000 MPa and a (*f*_a,y_) of 190 MPa. To govern the structural interactions at the meshed joint interface, a surface-to-surface contact pairs formulation was established. The outer helical surfaces of the fastening screw threads were designated as the contact surfaces using CONTA174 elements, while the corresponding internal thread walls of the RHS profile hole acted as the target surfaces via TARGE170 elements. The tangential contact behaviour was controlled by an isotropic Coulomb friction model with a constant friction coefficient specified as 0.3 to govern the mechanical interlocking. For the boundary conditions, the geometric constraints perfectly mirrored the experimental setup: all translational degrees of freedom were fully restricted at the supported nodes of the dual steel clamping frames, and a monotonic axial pulling displacement was progressively applied to the screw head along its longitudinal axis to drive the progressive pull-out simulation. To properly simulate the connection’s failure mechanism, a strain-based failure criterion was assigned to the screw threads in ANSYS. The failure initiation is governed by the critical equivalent plastic strain corresponding to the ultimate tensile strength of the screw material (*ε*_f_ = 0.053, where the true stress reaches its peak at 262 MPa). Elements exceeding this threshold lose their load-bearing capacity and are removed from the calculation, thereby successfully predicting the failure initiation and preventing unconstrained plastic flowing at the thread region.

### 3.3. Validation of Numerical Model

To guarantee the reliability of the established numerical framework, the FE simulation results were rigorously validated against the experimental benchmarks from both macroscopic and microscopic perspectives. Macroscopically, as depicted in the *F*-*δ* comparisons in Figure 5, the numerical curves (denoted by the green circular markers) track the multi-stage experimental trajectories with high fidelity. The FE model successfully replicates the distinct initial adaptation phase under Failure Mode I (Figure 5a) as well as the linear-elastic dominance under Failure Mode II (Figure 5b). Quantitative assessments indicate that the discrepancy in predicting *F*_u_ between the physical tests and the numerical simulations remains strictly within 5%, demonstrating excellent predictive accuracy.

To rigorously quantify the accuracy and fit quality of the established finite element model, a comprehensive statistical evaluation was performed across all specimen series. Specifically, the root mean square error (RMSE), the coefficient of determination (R^2^), the relative error for *K*_u_, and the error for displacement corresponding to the maximum force *δ*_u_ were systematically calculated. As compiled in Table 2, the R^2^ values for the entire loading trajectories across all specimens exceed 0.97, and the average RMSE remains at a remarkably low level (below 0.25 kN). Furthermore, the relative discrepancies in predicting the *K*_u_ and the *δ*_u_ are strictly bounded within 2% and 4.6%, respectively. This multi-indicator quantitative validation firmly establishes the high fidelity and mathematical reliability of our numerical framework.

Microscopically, the simulated structural responses provide an in-depth mechanical explanation for the observed failure modes. As illustrated by the overall connection stress contours in Figure 6c and the detailed component stress fields in Figure 6d, the intense stress concentration zone highlighted in red is highly localised at the immediate contact interface between the primary screw threads and the internal hole walls of the aluminium RHS profile. The von Mises stress swiftly propagates through the first two to three engaged thread pitches, exceeding the material yield strength of the 6063-T6 aluminium alloy. This localised yielding behaviour captures the physical essence of the thread stripping and shear peeling mechanism characteristic of Failure Mode I. Simultaneously, for specific geometric configurations, the stress contours along the screw shank beneath the head approach its ultimate strength limits, which perfectly aligns with the tensile fracture mechanism categorised in Failure Mode II. Consequently, the high conformity in both macro curves and micro failure behaviours establishes a solid foundation for subsequent stochastic assessments.

To eliminate mesh dependency and verify numerical reliability, a rigorous mesh convergence analysis was systematically executed. Four distinct mesh density levels were evaluated by varying the local element size at the thread engagement zone from 1.2 mm to 0.2 mm, yielding total element counts from approximately 48,000 to 320,000. As illustrated in Figure 6e, when the local mesh size was refined below 0.4 mm, the variation in the calculated ultimate pull-out capacity *F*_u_ and the peak Von Mises stress dropped below 1.1%, demonstrating stable mesh independence. Balancing computational efficiency and localised contact fidelity, a local mesh size of 0.4 mm was strictly implemented for the thread interfaces across all models.

## 4. Reliability and Parameter Analysis

### 4.1. Framework of LHS–RSM Combined Stochastic Simulation

To bypass the prohibitive computational costs traditionally associated with direct large-scale non-linear finite element simulations, a multi-stage stochastic assessment framework combining Latin Hypercube Sampling (LHS) and Response Surface Methodology (RSM) was implemented within the ANSYS Probabilistic Design System (PDS). The operational workflow of this advanced simulation scheme proceeds through a well-defined sequential loop. Initially, a comprehensive set of critical material properties and geometric parameters governing both the fastening screw and the aluminium RHS profile are optimised and selected as probabilistic input random variables. Based on the verified deterministic FE model established in Section 3, the LHS technique is executed to partition the multi-dimensional parameter space, generating a precise set of representative stochastic sample points. These discrete sample points are then evaluated via automated batch-executed random FE simulations to capture the corresponding non-linear pull-out responses. Subsequently, the discrete input–output datasets gathered from the random FE simulation samples are utilised to construct a high-precision metamodel. The RSM smoothly interpolates the complex, implicit structural behaviours into explicit polynomial response surfaces that map the ultimate limit states. Armed with these computationally efficient mathematical expressions, a classic large-scale Monte Carlo Simulation (MCS) loop consisting of tens of thousands of random trials is performed. This dual-method strategy allows for the comprehensive propagation of parameter uncertainties to obtain robust statistical populations under negligible computational overhead. Finally, the aggregated stochastic datasets are processed to output quantified scatter distributions, multi-dimensional boundary profiles, and global sensitivity coefficients, establishing a rigorous basis for structural assessment under construction overloads.

### 4.2. Statistical Distributions of Inputs and Representative Output Sample Curves

To execute the stochastic simulations within the ANSYS PDS environment, the uncertainties inherent in both material manufacturing and geometric tolerances must be reasonably propagated. In alignment with engineering practice and design code requirements for solar PV racking components, all input random variables, including the material indices (*E*_a_ and *E*_s_), the fastening screw variables (*p* and *D*), and the RHS structural dimensions consisting of the wall thickness (*t*_0_) and section width (*b*_0_), were mathematically defined following the classical Gaussian normal distribution. Specifically, the coefficients of variation (COV) for the geometric variables (*t*_0_, *b*_0_, *p*, and *D*) were strictly controlled between 1% and 2% in accordance with high-precision Computer Numerical Control (CNC) machining lines, while the COVs for the material properties (*E*_a_ and *E*_s_) were assigned as 5% and 4%, respectively, mirroring the statistical scattering observed in the baseline experimental repetitions.

The adoption of Gaussian distributions for these variables is firmly rooted in the Central Limit Theorem (Law of Large Numbers). Under automated mass-production and standard fabrication environments, geometric deviations and material variations are driven by a multitude of microscopic, mutually independent random factors; their cumulative effect inherently asymptotes to a normal probability distribution. This approach is highly consistent with standard structural safety authority recommendations, such as the Joint Committee on Structural Safety (JCSS) [18] and standard industrial tolerancing guidelines (e.g., ISO 2768 [19]).

**Figure 8:**
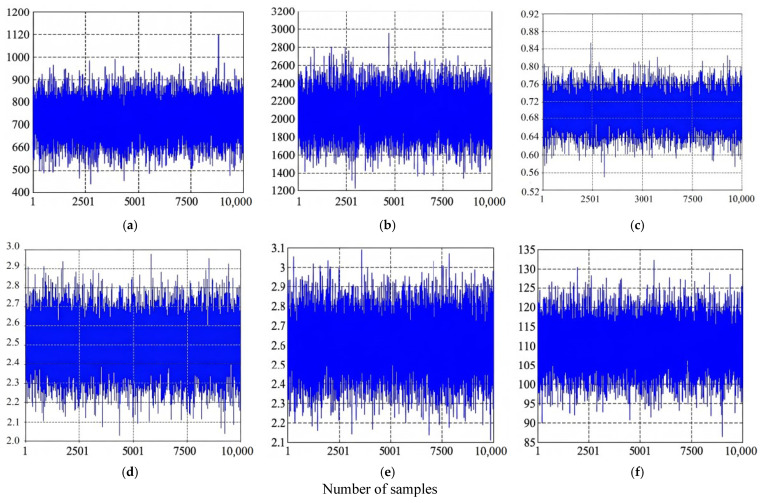
Simulation sample curves for (**a**) (×100) *E*_a_; (**b**) (×100) *E*_s_; (**c**) *p*; (**d**) *D*; (**e**) *t*_0_; (**f**) *b*_0_.

**Figure 9:**
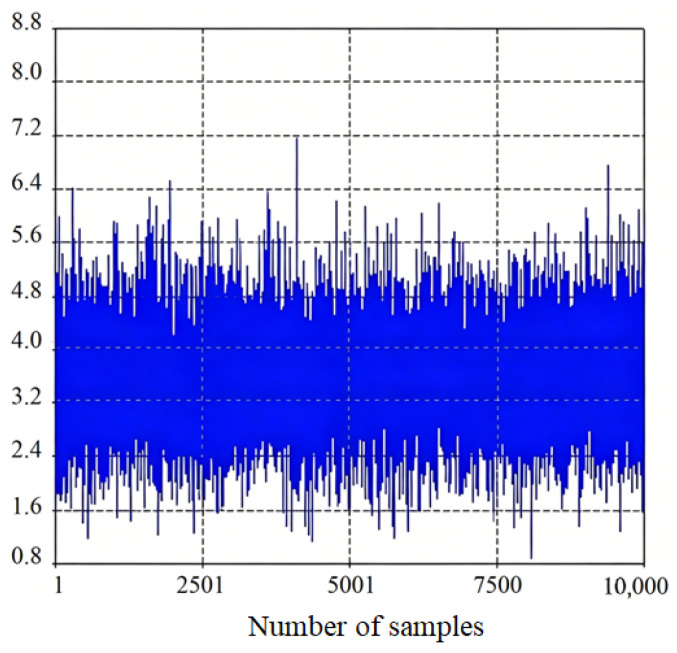
Simulation sample curves for *F*_r_.

The generation of these input populations over a large-scale simulation scope of 10,000 iterations is sequentially illustrated in Figure 8a–f. The fluctuating sample history bands effectively demonstrate that the LHS method populates the predetermined multi-dimensional parameter space uniformly without localised cluster gaps, matching the statistical characteristics of the high-order normal probability density functions. The representative output sample curve representing the structural safety limit state is captured in Figure 9. In this analysis, the target behavioural indicator *F*_r_ is defined as the random elastic pull-out capacity of the fastening screw joint. While the experimental program in Section 2 targeted the ultimate limit state to uncover the progressive post-yield failure mechanisms (such as thread stripping), transferring the same ultimate capacity directly to the probabilistic reliability execution would be overly non-conservative for solar structural safety. Evaluating *F*_r_ instead of the post-yield ultimate limit state perfectly aligns with the stringent structural design requirements for high-rise roof PV systems, where any localised micro-yielding or permanent plastic loosening under wind vibrations is strictly prohibited. Thus, the experimental findings serve as a benchmark to calibrate the model’s accuracy, while the subsequent stochastic loop shifts the focus to the elastic serviceability threshold to ensure operational zero-plasticity redundancy under dynamic wind fatigue environments. As shown in the simulation history plot in Figure 9, the generated population of *F*_r_ exhibits a highly responsive stochastic fluctuation ranging primarily between 1.6 kN and 5.6 kN, driven by the random combinations of inputs. This extensive history distribution confirms that, even within the elastic regime, the load-bearing performance of the fastening screw connection is highly susceptible to small geometric and material uncertainties, which highlights the critical engineering necessity for the multi-dimensional response surface analysis in the next section.

### 4.3. Multi-Dimensional Response Surface Characterisation

In the structural design of fastening screw connections for aluminium alloy PV support structures, the primary focus is predominantly directed toward *F*_r_ rather than the deformation limits, with deformation serving as a secondary consideration. This design hierarchy is directly validated by the experimental failure mechanisms observed in Section 2.3. Structural failure, particularly via Failure Mode I, typically manifests as sudden thread stripping and localised profile tearing at the joint interface. Because this catastrophic detachment occurs abruptly once the resistance threshold is breached, guaranteeing adequate *F*_r_ is paramount to ensuring structural survivability, whereas the corresponding elastic displacement *δ*_r_ primarily functions to monitor structural serviceability under non-destructive loading regimes. To thoroughly map the non-linear interactive dependencies of these performance indicators, three-dimensional (3D) response surfaces were constructed across three sequential evaluation stages.

**Figure 10:**
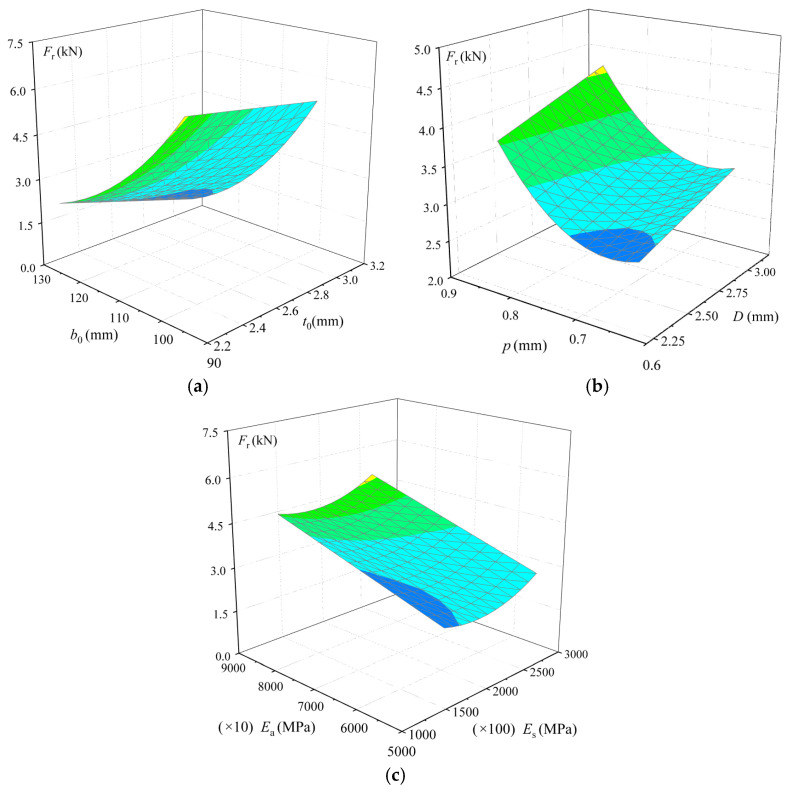
Illustration of response surfaces for: (**a**) *F*_r_–*b*_0_–*t*_0_, (**b**) *F*_r_–*D*–*p*, (**c**) *F*_r_–*E*_a_–*E*_s_.

The first evaluation stage focuses on the direct relationship between *F*_r_ and the three major categories of design parameters: RHS geometric configurations, fastening screw geometries, and baseline material properties. Figure 10a illustrates the coupled effects of the RHS structural dimensions *b*_0_ and *t*_0_ on *F*_r_. The response surface exhibits a highly non-linear, concave exponential upward trend along the *t*_0_ axis, revealing that increasing the wall thickness from 2.2 mm to 3.2 mm yields a dramatic expansion in *F*_r_ from approximately 2.0 kN to over 6.0 kN due to the direct expansion of the thread engagement cylinder, while variations in *b*_0_ from 90 mm to 130 mm render an almost horizontal, negligible effect. Figure 10b delineates the interaction between the fastening screw parameters *D* and *p* and *F*_r_. The topology presents a characterised twisted saddle-like surface, where a prominent peak zone exceeding 4.5 kN is generated exclusively at the convergence boundary of the maximum diameter (*D* = 3 mm) and minimum pitch (*p* = 0.60 mm), capturing a profound geometric coupling effect. Figure 10 depicts the relationship between the material parameters *E*_a_ and *E*_s_ and *F*_r_, demonstrating that *E*_a_ dominates the initial slope of the strength domain with a distinct linear inclination ranging between 50,000 MPa and 90,000 MPa, whereas *E*_s_ within the range of 100,000 MPa to 300,000 MPa provides secondary stabilisation.

**Figure 11:**
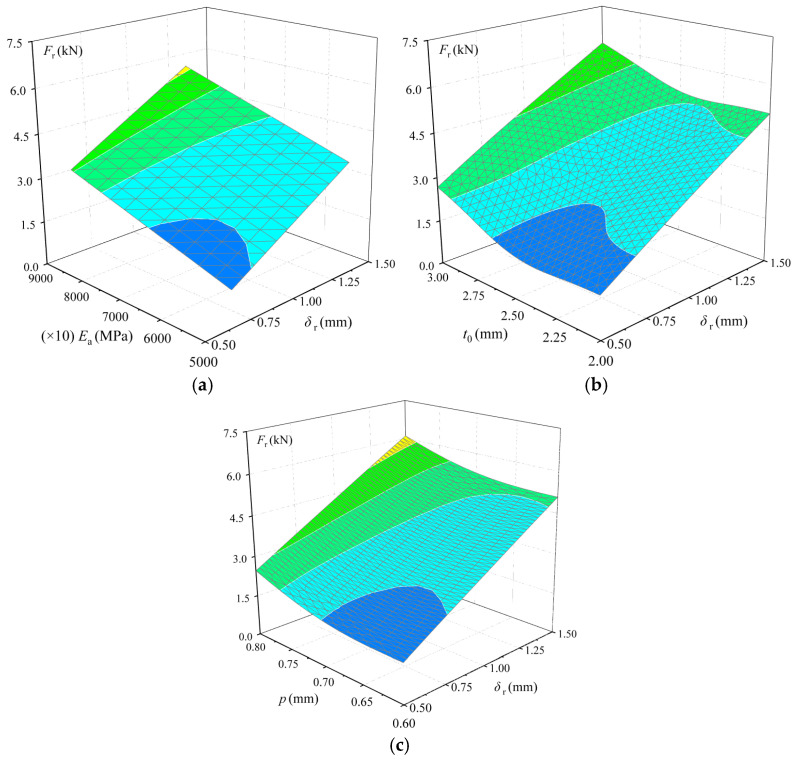
Illustration of response surfaces for: (**a**) *F*_r_–*E*_a_–*δ*_r_, (**b**) *F*_r_–*t*_0_–*δ*_r_, (**c**) *F*_r_–*p*–*δ*_r_.

The second evaluation stage investigates the multi-dimensional correlation among *F*_r_, *δ*_r_, and the most dominant parameter extracted from each of the three major categories. This characterisation is compiled across Figure 11a–c, representing the *F*_r_–*E*_a_–*δ*_r_, *F*_r_–*t*_0_–*δ*_r_, and *F*_r_–*p*–*δ*_r_ spatial configurations, respectively. In all three topologies, the gradient along the *δ*_r_ axis is exceptionally steep, which indicates that, within the purely elastic regime bounded by *δ*_r_ ≤ 1.5 mm, the mobilisable tensile resistance increases rapidly with minor increments in displacement. Specifically, Figure 11a,b demonstrate that, at higher *δ*_r_ boundaries approaching 1.50 mm, maximising *E*_a_ to 90,000 MPa and *t*_0_ to 3 mm drastically scales up the peak value of *F*_r_ to its global maximum of 7.5 kN, further verifying that the stiffness matching of the connection is heavily constrained by the structural profile properties rather than the fastener elasticity. Conversely, the *F*_r_–*p*–*δ*_r_ surface in Figure 11c highlights that a finer thread pitch approaching 0.6 mm delays localised micro-slipping, allowing the joint to mobilise a higher *F*_r_ at smaller elastic deformations below 0.75 mm.

**Figure 12:**
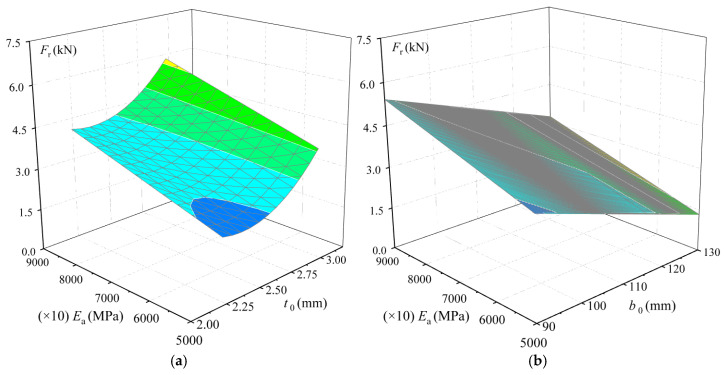
Illustration of response surfaces for: (**a**) *F*_r_–*E*_a_–*t*_0_, (**b**) *F*_r_–*E*_a_–*b*_0_.

The third evaluation stage directs specific attention to the behavioural constraints of the RHS profile wall by mapping *F*_r_ against the aluminium material index *E*_a_ and the cross-sectional geometry variables *t*_0_ and *b*_0_. As shown by the response surface profiles in Figure 12a,b, which characterise the *F*_r_–*E*_a_–*t*_0_ and *F*_r_–*E*_a_–*b*_0_ interfaces, the non-linear coupling effects are highly pronounced. In Figure 12a, the concurrent expansion of *E*_a_ towards 90,000 MPa and *t*_0_ towards 3 mm generates a highly synchronised, sweeping diagonal slope that yields the global maximum value of *F*_r_ near 7.5 kN. In contrast, the *F*_r_–*E*_a_–*b*_0_ surface in Figure 12b exhibits a uniform, two-dimensional linear tilt with minimal curvature, maintaining a stable capacity plateau across the *b*_0_ variations from 90 mm to 130 mm. This tailored mapping directly corresponds to the physical nature of Failure Mode I, which represents the dominant failure condition under most real-world solar racking operations. The spatial profiles demonstrate that optimising *E*_a_ in tandem with *t*_0_ creates a highly reliable structural synergy that effectively suppresses localised flange flexing and out-of-plane micro-tearing around the screw hole, thereby ensuring that the fastening screw joint maintains structural integrity under extreme uplift actions.

### 4.4. Scatter Distributions and Global Sensitivity Quantifications

**Figure 13:**
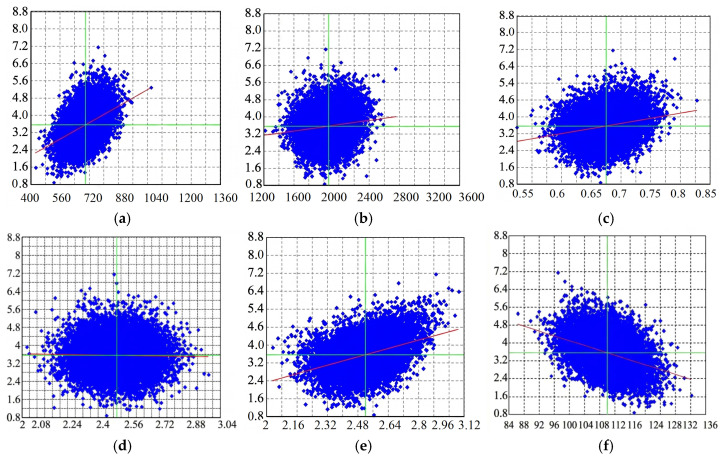
Scatter plots for *P*_k_ related to: (**a**) *E*_a_ (×10^2^), (**b**) *E*_s_ (×10^2^), (**c**) *p*, (**d**) *D*, (**e**) *t*_0_, (**f**) *b*_0_.

To visually characterise the probabilistic correlation between the individual stochastic inputs and the structural response, the large-scale simulation populations are projected via multi-dimensional scatter distributions, as compiled in Figure 13a–f. Each discrete parameter dataset is superimposed with an analytical linear regression tendency line to quantify the directional correlation and variance spread of *F*_r_. The numerical results demonstrate that *F*_r_ exhibits a highly pronounced, tight upward clustering tendency with respect to *E*_a_ and *t*_0_. Within the Gaussian variation band of *E*_a_ (40,000 MPa to 104,000 MPa) and *t*_0_ (2.16 mm to 3.12 mm), the positive slope of the regression line is exceptionally steep, confirming that higher material stiffness and an expanded thread engagement wall thickness provide a highly reliable baseline to upgrade the joint capacity. Conversely, a distinct negative correlation is revealed between *b*_0_ and *F*_r_ in Figure 13f, where the regression line slopes downward as *b*_0_ expands from 84 mm to 132 mm. This adverse mechanism indicates that widening the profile flange section under a constant thickness worsens local out-of-plane flexing under localised tension, slightly undermining the net pull-out resistance threshold. In sharp contrast, the scatter swarms for *E*_s_, *p*, and *D* display highly symmetric, localised circular distributions with nearly horizontal regression gradients, indicating a substantially lower direct marginal influence within the purely elastic regime.

**Figure 14:**
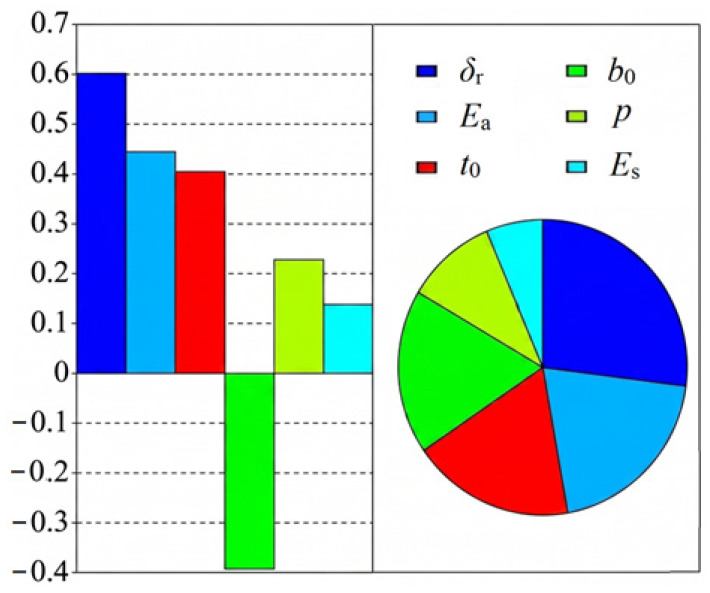
Comparison of sensitivities results for *F*_r_.

To provide a definitive, quantified comparison of these interactive parameters, the final global sensitivity analysis results for *F*_r_ are extracted and compiled in Figure 14 via a paired bar chart and a directional variance contribution pie chart. The sensitivity index evaluations reveal that the elastic displacement *δ*_r_ yields the highest dominant positive sensitivity coefficient of approximately 0.60. This statistical dominance highlights that, within the strict boundary of the elastic state, the mobilisable pulling capacity is primarily driven by the progressive deformation development. Among the material and intrinsic geometric parameters of the assembly, *E*_a_ and *t*_0_ emerge as the primary controlling variables, exhibiting high positive sensitivity coefficients of approximately 0.44 and 0.40, respectively. These quantified levels underscore that the elastic limit of the connection is predominantly governed by the stiffness and local shear area of the 6063-T6 aluminium profile. Furthermore, the global evaluation confirms a pronounced negative sensitivity coefficient for *b*_0_ at approximately −0.39, which quantitatively establishes that an excessive flange section width induces local out-of-plane compliance that actively degrades the net connection efficiency. Regarding the fastening screw configurations, the thread pitch *p* maintains a moderate positive sensitivity index of 0.23, whereas the steel elastic modulus *E*_s_ provides the minimal structural contribution at approximately 0.14. It is worth noting that *D* registers a negligible statistical footprint in the final global ranking, indicating that, once the geometric failure classification is established, minor manufacturing variations in fastener thickness do not alter the elastic resistance domain. Consequently, these sensitivity vectors dictate that, to effectively optimise the safety of fastening screw connections in aluminium alloy PV support structures against severe uplift actions, structural designers must prioritise increasing the RHS flange wall thickness *t*_0_ and specifying high-grade profiles over modifying fastener material properties.

The negligible sensitivity footprint of the nominal shank diameter (*D*) within the elastic limit domain can be explained by the localised stress concentration mechanism shown in Figure 6. Once Failure Mode I (thread stripping) is established, the structural capacity is heavily dictated by the localised shear resistance of the thin-walled aluminium internal thread cylinder rather than the body thickness of the steel screw fastener itself. For the pronounced negative effect of the RHS section width (*b*_0_), the physical mechanism lies in the cross-sectional boundary constraints under localised tension. Widening the profile flange while keeping the thickness constant drastically reduces the out-of-plane flexural stiffness of the RHS upper plate. Under the intense localised axial pull applied by the screw, a larger *b*_0_ promotes severe out-of-plane rotational deformability and plate flexural bending around the screw hole. This localised flexing induces a severe uneven stress distribution and premature microscopic prying/peeling at the first engaged thread pitch, thereby degrading the net elastic pull-out resistance threshold. Regarding generalisability, these coupled physical mechanisms and non-linear response trends are fundamental to thin-walled structures and can be reasonably extended to other common configurations (e.g., L-shaped or open channel profiles), provided that the failure is governed by localised base metal tearing under localised point loading.

## 5. Conclusions

This paper presents a comprehensive experimental, numerical, and probabilistic investigation into the tensile pull-out performance and parameter sensitivities of fastening screw connections in aluminium alloy PV support structures under wind-induced uplift actions. A series of static pull-out tests were first conducted to classify the macro failure mechanisms and characterise the force–displacement (*F*-*δ*) relationships. Subsequently, high-fidelity 3D finite element models incorporating explicit thread geometries were developed in ANSYS and validated against experimental benchmarks with an ultimate load error within 5%. Finally, to circumvent excessive computational costs, a multi-stage simulation loop integrating Latin Hypercube Sampling (LHS) and Response Surface Methodology (RSM) was implemented within a large-scale Monte Carlo framework. This advanced stochastic assessment successfully decoupled the interactive configurations and quantified the global sensitivity profiles under the elastic limit state. Based on the comprehensive findings, the following major conclusions can be drawn:

- The mechanical response of the connection is strictly governed by the relative stiffness coupling between the fastening screw and the profile RHS wall. The connections are failed via two distinct failures: mode I (thread stripping and base metal tearing), characterised by severe internal thread peeling and a pronounced initial adaptation/seating regime on the *F*-*δ* curve, and mode II (screw shank torsional-tensile fracture), characterised by brittle rupture and linear-elastic dominance.
- The established finite element framework successfully bypasses the limitation of conventional simplified models that rely on artificial friction factors. The simulated micro-stress contours precisely capture the progressive yielding and peeling nature at the direct contact zone of the first two to three engaged thread pitches, providing a reliable numerical tool for predicting complex micro-thread boundary interactions.
- The global stochastic sensitivity assessment under the elastic regime establishes that the mobilisable elastic capacity *F*_r_ is predominantly dictated by the allowed deformation boundary (*δ*_r_ sensitivity index is nearly 0.60). Among the material and cross-sectional inputs, the aluminium elastic modulus *E*_a_ and the RHS wall thickness *t*_0_ emerge as the primary controlling variables with high positive sensitivities of 0.44 and 0.40, respectively, demonstrating that profile properties heavily constrain the elastic baseline.
- The global evaluation reveals a pronounced negative sensitivity coefficient for the RHS section width (b0 is nearly 0.39), demonstrating that expanding the flange width under a constant thickness promotes out-of-plane compliance that degrades net joint efficiency. Conversely, the fastener diameter D displays a negligible marginal footprint in the elastic domain. Therefore, to optimise the safety of aluminium alloy PV support structures against extreme uplift suction, engineers should prioritise upgrading the profile thickness t0 and material grade over modifying fastening screw dimensions.

Although the established deterministic and stochastic frameworks provide accurate predictions for static pull-out capacity, certain limitations regarding the generalisability of the results to long-term operational environments must be acknowledged. This study primarily simulated quasi-static monotonic loading regimes. In real-world engineering service conditions, high-rise roof PV support structures are inevitably subjected to high-cycle aerodynamic fatigue, cyclic wind reversals, and severe atmospheric environmental degradation (e.g., thermal expansion mismatch or galvanic corrosion). These dynamic and environmental factors may accelerate micro-crack propagation and thread loosening at the interlocking interfaces, potentially degrading the long-term pull-out resistance. Future investigations will incorporate cyclic fatigue boundary conditions and accelerated corrosion aging tests to further refine the global lifecycle reliability framework of these thin-walled connections.

## Figures and Tables

**Table 1:** List of geometric parameters for crews and test results.

Specimen	Screw Parameters					Test Results			
	*D*	*p*	dh	th	*F*_u_	COV(*F*_u_)	*δ*_u_	*K*_u_	Failure Mode
	(mm)				kN	%	mm	N/mm	
MS-D5P0.8	4.92	0.8	9.3	3.5	6.32	2.5	0.80	7885	I
MS-D5P1.1	4.92	1.1	9.4	3.4	6.71	3.8	1.67	4010	I
SS-D5P1.6	4.92	1.6	9.0	2.8	7.34	1.8	1.83	4022	I
MS-D6P1.3	6.26	1.3	12.2	4.4	6.69	4.1	2.48	5155	I
MS-D6P1.1	5.82	1.1	11.7	4.4	6.32	4.2	1.32	5247	I
MS-D8P1.3	7.90	1.3	15.7	5.9	8.00	2.4	3.77	2120	I
SS-D3P1.3	3.58	1.3	6.8	2.7	4.36	3.5	1.87	2384	II
SS-D4P1.3	4.18	1.3	7.8	2.8	6.92	1.5	1.41	4387	II

**Table 2:** List of exact statistical indicators related to test results.

Specimen	*F*_u,Exp_	*F*_u,Modelling_	Error (*F*_u_)	*δ*_u,Exp_	*δ*_u,Modelling_	Error (*δ*_u_)	Error (*K*_u_)	R^2^	RSME
	kN			mm					kN
MS-D5P0.8	6.32	6.45	2.13%	0.80	0.83	3.62%	1.44%	0.985	0.15
MS-D5P1.1	6.71	6.96	3.71%	1.67	1.72	2.81%	0.88%	0.978	0.21
MS-D6P1.3	6.69	6.84	2.24%	2.48	2.54	2.42%	0.17%	0.984	0.16
MS-D6P1.1	6.32	6.48	2.53%	1.32	1.38	4.55%	1.93%	0.977	0.24
SS-D3P1.3	4.36	4.45	2.09%	1.87	1.91	2.14%	0.05%	0.983	0.10
SS-D4P1.4	6.92	7.11	2.75%	1.41	1.44	2.49%	0.25%	0.977	0.21

## Data Availability

The original contributions presented in this study are included in the article. Further inquiries can be directed to the corresponding authors.
